# Long-term outcomes of pelvic organ prolapse repair using a mesh-capturing device when comparing single- versus multicenter use

**DOI:** 10.1007/s00404-020-05764-3

**Published:** 2020-09-11

**Authors:** Christian Falconer, Daniel Altman, Georgios Poutakidis, Päivi Rahkola-Soisalo, Tomi Mikkola, Edward Morcos

**Affiliations:** 1 Department of Clinical Sciences, Danderyd Hospital, Karolinska Institutet, 171 77 Stockholm, Sweden; 2 Department of Obstetrics and Gynecology, Danderyd Hospital, 182 88 Stockholm, Sweden; 3 Department of Women’s and Children’s Health, Uppsala University, Uppsala, Sweden; 4 Department of Obstetrics and Gynecology, University of Helsinki and Helsinki University Hospital, Helsinki, Finland; 5 Department of Obstetrics and Gynecology, University of Helsinki and Helsinki University Central Hospital, Folkhälsan Research Center, Helsinki, Finland

**Keywords:** Pelvic organ prolapse, Transvaginal mesh, Surgical volume, Safety, Effectiveness

## Abstract

**Purpose:**

The aim of this study was to compare long-term effects of high-volume surgery at a single-center to multicenter use when using a mesh-capturing device for pelvic organ prolapse (POP) repair.

**Methods:**

Five years after surgery 101 (88%) at the single center were compared with 164 (81.2%) in the multicenter trial. Outcome measurements included clinical examination, prolapse-specific symptom questionnaires [Pelvic Floor Distress Inventory 20 (PFDI-20), Pelvic Floor Impact Questionnaire—short form (PFIQ-7), Pelvic Organ Prolapse/Urinary Incontinence Sexual Questionnaire (PISQ-12)] and pain estimation by VAS (0–10).

**Results:**

Optimal apical segment outcome was 95% in the single- compared to 83.3% in the multicenter study (*p* < 0.001). POP recurrence in the anterior and posterior walls (POP-Q, Ba and Bp ≥ 0) was more common at the multicenter as compared to the single center [(19.8% vs 5.4%) and (26% vs 2.7%), (*p* < 0.001)]. Reoperations for POP and mesh-related complications were more frequent in the multicenter study [31/202 (15.3%) vs 7/116 (6.1%), *p* < 0.001]. Total PFDI-20, PFIQ-7 and PISQ-12 scores were comparable between the cohorts. There were no significant differences in overall pain scores in-between the cohorts during follow-up. At the single center, 1/81 patients (1.2%) had VAS 7/10, i.e. severe pain, as compared to 3/131 (2.3%) in the multicenter study (*p* = 0.277).

**Conclusions:**

Despite the high objective and subjective long-term effectiveness of the procedure in both regular use, and at a high-volume center, centralizing the use of a standardized capturing-device guided transvaginal mesh for POP repair reduced secondary interventions by more than half.

## Introduction

Pelvic reconstructive surgery using the transvaginal Uphold™ Lite mesh kit to suspend the apical vaginal segment has been shown to effectively ameliorate pelvic organ prolapse (POP) symptoms and restore anatomical outcomes at short term [1–8]. Due to a lack of “sufficient evidence to assure that the probable benefits of the devices outweigh their probable risks”, the FDA decided that vaginal mesh products should not be distributed in the US (FDA 2019) [9]. As a consequence, many mesh manufacturers discontinued manufacturing and sales of the products worldwide. However, there is a continued need for investigating safety and effectiveness of transvaginal mesh devices since many patients who have already undergone the procedures are facing unknown long-term outcomes.

It is well established that centralization of complex or high-risk procedures to high-volume centers may optimize outcomes, increase patient safety, decrease mortality and also increase cost-effectiveness [10–18]. In the field of pelvic reconstructive surgery, there is, however, scarce evidence to support this claim. As a consequence, it is not known how long-term outcomes and morbidity may differ between the settings [2, 4, 8]. Increased knowledge on the differences in outcomes between single- and multi-center use of surgical innovations in urogynecology may guide and improve the clinical introduction of new surgical procedures in the future. The aim of this study was to evaluate if high-volume center outcomes are superior to the results of regular use of Uphold mesh kit for apical prolapse at long term and how this relates to long-term morbidity of the procedure.

## Materials and methods

For the purpose of the study, we combined data derived from two separate studies designed as a single- and a multi-center study [2, 4, 8]. These studies aimed to evaluate various clinical aspects of the Uphold mesh kit. All patients included in this study had symptomatic and quantified apical segment (uterine or vaginal vault) prolapse ≥ stage 2, with or without anterior vaginal wall prolapse, according to the pelvic organ prolapse quantification (POP-Q) system [19]. Anatomical outcomes were assessed using the POP-Q system during a gynecological examination with the patient in a lithotomy position at which time mesh exposure or other healing defects were recorded. POP-Q stage 0 or 1 in the apical compartment was considered an optimal anatomical outcome and was the primary outcome measure for the analysis.

Using a standardized procedure with a capturing device, a monofilament, macroporous and uncoated polypropylene mesh (Uphold™ Lite) was placed to suspend the apical vaginal segment [2, 4]. All patients at the single center were operated by two experienced urogynecological surgeons (50 and 65 patients, respectively) at the Department of Obstetrics and Gynecology, Danderyd Hospital, Stockholm, Sweden.

The multicenter study included a total of 207 patients operated by 26 surgeons at senior consultant level throughout 24 centers in Sweden, Finland, Denmark and Norway [2, 8]. Data have been previously published and described in detail [2, 8]. The 24 centers were located as 11 centers at Sweden, 4 centers at Finland, 5 centers at Norway and 4 centers at Denmark. The rate of surgery was from 1 to 13 patients for each surgeon (mean 8 patients/surgeon) and 1–24 patients for each center (mean 8.6 patients/center).

The study protocol was near identical for both studies and neither study was blinded [2, 4, 8]. Exclusion criteria for both studies included: current or previously treated pelvic organ cancer, cervical elongation, severe rheumatic disease, insulin-treated diabetes mellitus, connective tissue disorders, current systemic steroid treatment, and urinary incontinence. Follow-up after surgery was performed after 1 and 5 years in the multicenter study and 2 and 5 years in the single-center study. There were no restrictions on weight, parity, menopausal status, or previous surgery although urinary incontinence was not an exclusion criterion at the single center. In both studies, patients were included by the responsible urogynecologist if a patient fulfilled the inclusion and no exclusion criteria.

Subjective disease-specific pelvic floor outcomes were assessed by the Pelvic Floor Distress Inventory 20 (PFDI-20) questionnaire which includes 3 scales of 20 questions: Urinary Distress Inventory-6 (UDI-6), Pelvic Organ Prolapse Distress Inventory-6 (POPDI-6), and Colorectal-Anal Distress inventory-8 (CRADI-8) [20]. The Pelvic Floor Impact Questionnaire—short form (PFIQ-7) was used to assess effects on quality of life [20]. The PFIQ-7 includes the Urinary Impact Questionnaire (UIQ-7), the Colorectal-Anal Impact Questionnaire (CRAIQ-7) and the Pelvic Organ Prolapse Impact Questionnaire (POPIQ-7) [20]. To measure the impact on patients’ sexual function, Pelvic Organ Prolapse/Urinary Incontinence Sexual Questionnaire (PISQ-12) was used which includes 12 questions concerning behavioral/emotional, physical and partner related sexual domains [21]. Additional surgical procedures, POP recurrence, as well as, interventions for mesh-related complications following the primary operation was registered up to 5 years after surgery.

A Visual Analogue Scale (VAS) was used to assess pelvic pain at baseline and follow-up visits [22]. The 11-point scale ranges from 0 to 10 where 0 indicates no pain and 10 indicates maximal severe pain. In a sub-analysis of pain, we used the Numeric Rating Scale (NRS). The NRS for pain is classified into four categories; 0 indicates no pain, 1–3 mild pain, 4–6 moderate pain, whereas 7–10 indicate severe pain [22].

### Statistical analysis

Statistical analysis was performed using the predictive analysis software (IBM^@^SPSS^©^ Statistics, Version 25, Inc, Chicago, IL, USA, 2017). We used independent sample t tests to compare independent variables. The statistical difference between nominal data was tested by Chi-square test of independence. Repeated measures ANOVA was used to test total values and to compare continued variables and over time of follow-up between the single and the multicenter. The General Linear Model of analysis was used to compare changes over time and centers when analyzing the NRS scale for pain. Inference for population proportion analysis was used to statistically compare between proportions in the two different populations, i.e. the single and multicenter. All missing data were considered as missing without imputation of data.

### Ethical approval

The studies were approved by the Stockholm Regional Ethical Review Board and ethic review committees in the respective countries as appropriate.

The single-center and the multicenter studies were registered at www.clinicaltrials.gov: NCT03077490 and NCT01823055 respectively.

## Results

Five years after surgery, 101/115 (88%) and 164/207 (79%) patients answered the patient-reported outcome questionnaires at the single and multicenter, respectively. 74/115 patients (64.3%) at the single-center and 139/207 patients (67.1%) at the multicenter trial were available for clinical examination and POP-Q evaluation.

Anatomical outcomes and POP-Q staging at 5 years after surgery are described in Table 1. An optimal anatomical apical segment outcome (POP-Q C stage 0–1) was achieved in 97.3% (72/74 patients) in the single-center cohort as compared to 97.5% (116/119) in the multicenter cohort (*p* = 0.938). If considering patients re-operated by any POP surgical procedure during follow-up as a failure, i.e. an optimal outcome (POP-Q stage 0–1 in the apical segment) and no secondary procedures the success rate was 95% (70/74) in the single-center and 83.3% (116/139) in the multicenter cohort (*p* < 0.001). When considering the anterior and posterior vaginal compartments separately, occurrence of anterior vaginal wall prolapse (POP-Q Ba ≥ 0) was 5.4% (4/74) in the single-center cohort vs. 19.8% (26/131) in the multicenter cohort (*p* < 0.001). For posterior vaginal wall prolapse (POP-Q Bp ≥ 0), the corresponding figures were 2.7% (2/74) vs. 26% (34/131) (*p* < 0.001). Other individual POP-Q items were largely comparable between the cohorts. There were no cases of mesh erosion (0/74) in the single-center study as compared to 3/139 (2.2%) in the multicenter study (*p* = 0.034).

**Table 1:** 5-year evaluation of anatomical outcomes following the Uphold procedure

POP-Q stage/compartment	Stage	Multicenter		Single center		*p* value
		*n* (%)	*n*	*n* (%)	*n*	
Apical (C)	0	45 (38%)	119	7 /10%)	74	0.938 [98% vs 97%
	1	71 (60%)		65 (88%)		(116/119) vs (72/74)]
	2	1 (1%)		1 (1%)		
	3	2 (2%)		1 (1%)		
	4	0		0		
Anterior wall (Aa)	0	52 (39%)	131	38 (51%)	74	0.002 [69% vs 92%
	1	41 (31%)		30 (41%)		(91/131) vs (67/74)]
	2	38 (28%)		4 (5%)		
	3	3 (2%)		2 (3%)		
	4	0 (0%)		0 (0%)		
Anterior wall (Ba)	0	38 (29%)	131	50 (68%)	74	< 0.001 [70% vs 91%
	1	53 (41%)		17 (23%)		(91/131) vs (67/74)]
	2	36 (28%)		3 (4%)		
	3	4 (3%)		4 (5%)		
	4	0 (0%)		0 (0%)		
Posterior wall (Ap)	0	19 (15%)	131	32 (43%)	74	< 0.001 [61% vs 92%
	1	61 (47%)		36 (49%)		(80/131) vs (68/74)]
	2	47 (36%)		5 (7%)		
	3	4 (3%)		1 (1%)		
	4	0 (0%)		0 (0%)		
Posterior wall (Bp)	0	33 (25%)	131	48 (65%)	74	< 0.001 [55% vs 89%
	1	39 (30%)		18 (24%)		(72/131) vs (66/74)]
	2	52 (40%)		6 (8%)		
	3	6 (5%)		2 (3%)		
	4	1 (1%)		0 (0%)		
Cystocele (Ba ≥ 0)		26 20%	131	4 5%	74	0. 005 [20% vs 5%]
Rectocele (Bp ≥ 0)		34 26%	131	2 3%	74	< 0.001 [26% vs 3%]
Pb (mean ± SD)		3.3 ± 0.9 (CI 3.2–3.5)	129	2.8 ± 0.7 (CI 2.7–3)	73	< 0.001
Gh (mean ± SD)		3.9 ± 0.9 (CI 3.8–4.1)	129	4 ± 1.0 (CI 3.8–4.3)	73	0.402

*p* value for apical, anterior and posterior wall compare success (POP-Q stage 0–1) vs failure (POP-Q stage 2–4). *p* < 0.05 was considered statistically significant (Chi-square test)

Total number of surgical interventions for pelvic floor insufficiency or mesh-related complications was 7/115 patients (6.1%) vs. 31/202 patients (15.3%) in the single- and multicenter cohorts, respectively, during the 5-years follow-up (*p* = 0.001). Four patients were re-operated because of postoperative pain (4/164, 2.4%) at the multicenter vs. none at the single center (*p* = 0.021). At the time of surgery, the mesh was removed in all four cases and in one case, a hysterectomy was performed.

There were no significant differences in demographic characteristics between the single and multicenter (Table 2). There were no significant differences between the cohorts in subjective outcomes, i.e. patient-reported symptoms as measured by the PFIQ-7, PFDI-20, PISQ-12 (Table 1). Although the UDI-6 domain score was higher in the single center (23.6 ± 19.61 vs. 17.2 ± 18.28, *p* = 0.01), indicating greater symptom severity of urinary incontinence, it did not significantly affect the total PFDI-20 score. The PISQ physical domain was significantly higher at the single center (17.7 ± 2.72 vs. 14.1 ± 3.48, *p* < 0.001), indicating higher sexual activity, whereas the PISQ-partner domain was lower at the single center (7.2 ± 2.51 vs. 10.09 ± 2.79, *p* < 0.001) indicating lower partner sexual activity. Neither of these differences affected the total PISQ-12 score.

**Table 2:** 5-year data comparing subjective outcomes in a single vs. multicenter study

	Multi-center			Single-center			*p* value
	Mean ± SD	CI	*n*	Mean ± SD	CI	*n*	
Age (years)	69.8 ± 11	68–71.7	138	70.8 ± 9.3	69.0–72.7	94	0.46
Weight (kg)	71 ± 10.3	69.1–73	110	70.8 ± 11	68.1–73.5	65	0.91
BMI	25.7 ± 5.9	24.6–26.8	111	26.1 ± 3.5	25.2–26.9	65	0.61
Pain (VAS scale)	0.4 ± 1.1	0.2–0.6	131	0.6 ± 1.38	0.2–0.9	81	0.29
PFIQ-7 total	13.7 ± 31.7	8.8–18.6	164	18.4 ± 32.8	11–25.7	79	0.29
PFIQ- UIQ7	6.4 ± 15.2	4.1–8.7	170	7.7 ± 14.2	4.5–10.1	80	0.52
PFIQ- CRAIQ7	4.4 ± 11.4	2.6–6.1	164	6.7 ± 14.3	3.5–9.9	79	0.17
PFIQ- POPIQ7	3.3 ± 10.1	1.7–4.8	166	4 ± 11	1.5–6.4	79	0.62
PFDI-20	47.4 ± 40.5	39.8–53.1	146	56.1 ± 44	45.5–66.7	69	0.11
POPDI-6	12.1 ± 15.2	9.7–14.5	156	15.9 ± 15.8	12.3–19.6	74	0.08
CRADI-8	14.4 ± 14.3	12.2–16.7	155	17.7 ± 17.6	13.6–21.8	73	0.12
UDI-6	17.2 ± 18.3	14.3–20.1	155	23.6 ± 19.6	19.2–28	78	0.01
PISQ-12 total	33.4 ± 8.1	31.4–35.4	66	34.6 ± 6.1	32.2–36.9	28	0.49
PISQ12-behavioral	8.3 ± 3.8	7.3–9.2	67	8.2 ± 4.2	6.8–9.6	35	0.95
PISQ-physical	14.1 ± 3.5	13.3–15	66	17.7 ± 2.7	16.7–18.6	35	< 0.001
PISQ- partner	10.09 ± 2.8	10.2–11.6	66	7.2 ± 2.5	6.3–8.1	31	< 0.001

Independent sample *t* test, *p* < 0.05 was considered statistically significant

In an analysis of changes over time, we performed pairwise comparisons of changes in reported pain outcomes and patient-reported pelvic floor questionnaires (PFIQ-7 and PFDI-20) if individual patient data were available at the two follow-up times (Table 3). There were no significant differences noticed when comparing changes in patient-reported outcomes between the cohorts. There was an insufficient number of pairwise observations with completed PISQ-12 scores to allow for a valid comparison between the cohorts over time.

**Table 3:** Pairwise comparison of changes over time

	Multi-center			Single-center			*p* value
	Mean ± SD	CI	*n*	Mean ± SD	CI	*n*	
Age (years)	3.9 ± 6.2	2.8 to 4.9	80	4.4 ± 1.8	4.0 to 4.7	78	0.440
Weight (kg)	0.9 ± 6.3	− 0.3 to 2.1	107	− 0.3 ± 3.6	− 1.2 to 0.6	64	0.176
BMI	0 ± 4.6	− 0.9 to 0.9	105	0 ± 1.4	− 0.4 to 0.3	63	0.924
Pain (VAS scale)	0 ± 0.5	− 0.1 to 0.1	106	0 ± 1.3	− 0.2 to 0.3	62	0.176
PFIQ- 7 total	− 12.70 ± 26	− 17 to − 8.2	131	− 7.5 ± 27.7	− 13.8 to − 1.1	76	0.190
PFIQ- UIQ7	− 3.2 ± 12.5	− 5.3 to − 1.1	138	− 1.9 ± 12	− 4.5 to 0.8	79	0.452
PFIQ- CRAIQ7	− 4.1 ± 9.93	− 5.8 to − 2.4	131	− 1.8 ± 12	− 4.5 to 0.9	77	0.145
PFIQ- POPIQ7	− 5.4 ± 7.38	− 6.7 to − 4.2	134	− 4.1 ± 9.7	− 6.3 to − 1.9	76	0.258
PFDI-20	2.3 ± 39.5	− 4.8 to 9.5	119	− 3.9 ± 35	− 12.5 to 4.7	66	0.284
POPDI-6	− 1.6 ± 20.1	− 5.2 to 1.9	126	− 1.4 ± 17.3	− 5.4 to 2.6	75	0.157
CRADI-8	0 ± 15.6	− 2.7 to 2.8	126	2.9 ± 15.1	− 0.7 to 6.5	70	0.220
UDI- 6	0.1 ± 16.3	− 2.8 to 2.9	127	− 3.3 ± 15.9	− 7.1 to 0.4	71	0.933

Independent sample t test was used for statistical analysis

Only patients with completed data collected at 1–2 years and 5 years after surgery were included in the analysis

Table 4 shows pain-related outcomes following surgery. The previously reported short-term improvements in patient reported pain was sustained at the 5-years follow-up after surgery in both cohorts (p < 0.001). Furthermore, there were no significant differences in estimated pain scores (VAS) in-between the cohorts during follow-up (independent-sample *t* test). At the single center, 1/81 patients (1.2%) had VAS 7/10, i.e. severe pain, as compared to 3/131 (2.3%) in the multicenter study (*p* = 0.277) based on the NRS scale.

**Table 4:** Analysis of pain before and after surgery

	Preoperative		Postoperative (1–2 years)		Postoperative (5 years)		*p* value
	Multicenter *n* = 190	Single-center *n* = 101	Multicenter *n* = 131	Single-center *n* = 101	Multicenter *n* = 131	Single-center *n* = 82	
VAS-scale (0–10)	1 ± 1.8 (CI: 0.8–1.3)	1.1 ± 2 (CI: 0.7–1.5)	0.3 ± 0.9 (CI: 0.2–0.5)	0.5 ± 1.3 (CI: 0.2–0.8)	0.4 ± 1.2 (CI: 0.2–0.6)	0.5 ± 1.2 (CI: 0.2–0.7)	< 0.001
NRS-scale							
No pain (0)	140 (74%)	66 (65%)	122 (93%)	84 (83%)	122 (92%)	65 (80%)	^#^
Mild pain (1–3)	35 (18%)	22 (22%)	8 (6%)	12 (12%)	7 (5%)	13 (16%)	
Moderate pain (4–6)	12 (6)	9 (9%)	0 (0%)	2 (3%)	0 (0%)	3 (4%)^¥^	
Severe pain (7–10)	3 (2%)	4 (4%)	1 (1%)	2 (2%)	3 (2%)	1 (1%)^**†**^	

ANOVA repeated measures was used for analysis of VAS-scale

^#^Using the general linear model of analysis, pain estimated by the NRS at both trials was significantly improved overtime (*p* = 0.001) and there was no significance between the two studies (*p* = 0.205)

^¥^One patient had complicated laparoscopic operation 3 years after primary surgery

^**†**^Patient had Paracetamol/Codeine treated low back pain at least 2 years before primary surgery with the mesh and continued with same treatment after surgery

## Discussion

In this 5-years analysis which combined data from two separate cohort studies using the Uphold^Lite^ mesh device for POP reconstructive surgery, we found that single-center high-surgery volumes resulted in less reoperations for POP recurrence and fewer mesh-related complications requiring surgical intervention. There were, however, no significant differences between the cohorts with regard to patient-reported outcomes. A number of studies have shown that the Uphold^Lite^ procedure for apical prolapse provide satisfactory subjective and objective outcomes and that high surgical volumes result in short-term decreased complication rates following apical mesh augmented surgery [1–8]. However, long-term efficacy and safety data have been missing.

Overall, we found that anatomical outcomes were sustainable over time with only minor, and non-significant, differences when comparing short and long-term follow-up both with regard to anatomical and patient-reported outcomes. When comparing the single- to the multi-center cohort 5 years after surgery, we also found no significant differences in our primary outcome (restoration of apical support) or in overall subjective scores with regard to condition specific symptoms, quality of life and sexual function. There was, however, a significant difference with regard to the occurrence of reoperations of both mesh-related complications and secondary pelvic reconstructive surgery, to the advantage of single-center use. The overall risk ratio for any complication in the multi-center compared to the single-center setting was 1.5:1 in patients undergoing the Uphold procedure as first-time prolapse surgery and 4:1 in patients undergoing surgery for recurrence in a previously operated compartment. These results suggest that a high surgical volume is beneficial for patients also at long term and that the risk for mesh-related complications requiring removal or revision of the mesh decreases with increased surgical experience. There is no universally accepted definition of high surgical volume and the number considered as high-volume certainly varies between different procedures. There is, however, a clear link between volume and patient morbidity, as well as, mortality in many fields. In concurrence with our results, a study by De Tayrac et al. showed a negative association between an increasing number of pelvic organ prolapse mesh procedures performed and the rate of complications [23]. Thus, emerging data suggest that the assumed relationship between volume and morbidity is valid also for urogynecological mesh surgery.

Subjective outcomes overall were improved also at long term when compared to baseline and largely sustained compared to the short-term outcomes. This was true also for perceived pain. Given recent years of widespread attention and highlighting of pain complications related to the use of vaginal mesh, it was somewhat surprising that levels of experienced pelvic pain in both the single- and multi-center cohort 5 years after surgery was low in general and still significantly lower than at baseline. We nonetheless recognize that long-term severe pain complications did occur and afflicted one and three patients in the single- and multi-center study, respectively. The mechanisms for mesh-related pain after biomaterial augmented surgery, although poorly understood, may be caused by a wide range of factors including different modes and routes of surgery, surgical dissection prior to placing the mesh, bleeding, infection, and pre-existing pain. The management of patients who experience persistent pain after mesh surgery remains clinically challenging and deserves further attention given our lack of knowledge on long-term consequences of mesh procedures, although many previously used products are no longer commercially available worldwide.

Three cases of mesh exposure were as reported in the multicenter cohort at the 5-years follow up, whereas none was reported in the single-center study. None of the three cases of mesh exposure reported persistent pain and were treated conservatively with topical estrogen without additional surgical interventions. Although suggested by our data, and supported by other studies [24–27], there was an insufficient number of cases to determine if high-volume single-center use was beneficial also with regard to postoperative mesh exposure at long term. Regardless, our long-term clinical data on more than 200 patients suggest that mesh exposure seems to be a relatively minor problem at long-term following this specific procedure and can be handled conservatively.

Despite early reports of risks and benefits associated with the use of vaginal mesh kits use of these products spread rapidly worldwide. In retrospect, one may argue that early centralization to specialized high-volume centers rather than widespread dissemination of mesh procedures could have prevented some complications and individual suffering. It would be inherently difficult to randomize patients between high-volume centers and clinics with lesser volumes due to geographic and social considerations. We must, therefore, rely on analyses from observational studies, such as the present one, to obtain information on the long-term effects of surgical volumes. Classification of the procedure was homogenous since all operations used an identical mesh kit performed in a standardized manner. There is an inevitable loss to follow-up when performing long-term clinical cohort studies. We believe that number of patients lost to follow-up in the present cohorts were not unusual with only minor differences between the single- and multi-center cohort suggesting a limited selection bias. A weakness of the study is that neither cohort was large enough to perform sub-analyses on possible predictors of both positive and negative outcomes with adequate statistical strength and precision. Factors such as pre-existing mood disorders, functional pain syndromes, smoking, obesity and previous pelvic surgery, may influence surgical outcomes but need further studies to understand the mechanisms involved [28, 29].

In summation, our data suggest that despite the high objective and subjective long-term effectiveness of the procedure in both regular use and at a high-volume center, centralizing the use of mesh kit procedures had clear clinical benefits. A reduction of secondary interventions by more than half when a standardized capturing-device guided transvaginal mesh was used at a high-volume center is an important advantage for patients and health care system alike. In countries where products similar to the one used in the present study remain in use, one may, therefore, advocate that mesh kit procedures should be centralized to high-volume center to minimize complications and secondary interventions.

## References

[1] Vu MK, Letko J, Jirschele K, Gafni-Kane A, Nguyen A, Du H, Goldberg RP (2012). Minimal mesh repair for apical and anterior prolapse: initial anatomical and subjective outcomes. Int Urogynecol J.

[2] Altman D, Mikkola TS, Bek KM, Rahkola-Soisalo P, Gunnarsson J, Engh ME, Falconer C, Nordic TVM group (2016). Pelvic organ prolapse repair using the Uphold™ Vaginal Support System: a 1-year multicenter study. Int Urogynecol J.

[3] Gutman RE, Rardin CR, Sokol ER, Matthews C, Park AJ, Iglesia CB, Geoffrion R, Sokol AI, Karram M, Cundiff GW, Blomquist JL, Barber MD (2017). Vaginal and laparoscopic mesh hysteropexy for uterovaginal prolapse: a parallel cohort study. Am J Obstet Gynecol.

[4] Morcos E, Altman D, Hunde D, Falconer C, Nordic TVM group (2018). Comparison of single- versus multicenter outcomes for pelvic organ prolapse repair using a mesh-capturing device. Int Urogynecol J.

[5] Lo TS, Pue LB, Tan YL, Hsieh WC, Kao CC, Uy-Patrimonio MC (2019). Anterior-apical single-incision mesh surgery (uphold): 1-year outcomes on lower urinary tract symptoms, anatomy and ultrasonography. Int Urogynecol J.

[6] Allegre L, Debodinance P, Demattei C, Fabbro Peray P, Cayrac M, Fritel X, Courtieu C, Fatton B, de Tayrac R (2019). Clinical evaluation of the Uphold LITE mesh for the surgical treatment of anterior and apical prolapse: a prospective, multicentre trial. Neurourol Urodyn.

[7] Gillor M, Langer S, Dietz HP (2019). A long-term comparative study of Uphold™ transvaginal mesh kit against anterior colporrhaphy. Int Urogynecol J.

[8] Rahkola-Soisalo P, Mikkola TS, Altman D, Falconer C, Nordic TVM Group (2019). Pelvic organ prolapse repair using the uphold vaginal support system: 5-Year follow-up. Female Pelvic Med Reconstr Surg..

[9] FDA 2019: https://www.fda.gov/news-events/press-announcements/fda-takes-action-protect-womens-health-orders-manufacturers-surgical-mesh-intended-transvaginal

[10] Filmann N, Walter D, Schadde E, Bruns C, Keck T, Lang H, Oldhafer K, Schlitt HJ, Schön MR, Herrmann E, Bechstein WO, Schnitzbauer AA (2019). Mortality after liver surgery in Germany. Br J Surg.

[11] Aikoye A, Harilingam M, Khushal A (2015). The impact of high surgical volume on outcomes from laparoscopic (totally extra peritoneal) inguinal hernia repair. J Clin Diagn Res.

[12] Chan JK, Gardner AB, Taylor K, Blansit K, Thompson CA, Brooks R, Yu X, Kapp DS (2015). The centralization of robotic surgery in high-volume centers for endometrial cancer patients–a study of 6560 cases in the US. Gynecol Oncol.

[13] Vanthoor J, Thomas A, Tsaur I, Albersen M, in collaboration with the European Reference Network for rare urogenital diseases and complex conditions (eUROGEN) (2019). Making surgery safer by centralization of care: impact of case load in penile cancer. World J Urol.

[14] Paraskevas KI (2019). The effect of centralization of abdominal aortic aneurysm repair procedures on perioperative outcomes. Ann Transl Med.

[15] Tol JA, van Gulik TM, Busch OR, Gouma DJ (2012). Centralization of highly complex low-volume procedures in upper gastrointestinal surgery. A summary of systematic reviews and meta-analyses. Dig Surg.

[16] Fisher AV, Ma Y, Wang X, Campbell-Flohr SA, Rathouz PJ, Ronnekleiv-Kelly SM, Abbott DE, Weber SM (2019). National trends in centralization of surgical care and multimodality therapy for pancreatic adenocarcinoma. J Gastrointest Surg.

[17] Lundström NR, Berggren H, Björkhem G, Jögi P, Sunnegârdh J (2000). Centralization of pediatric heart surgery in Sweden. Pediatr Cardiol.

[18] Markar SR, Karthikesalingam A, Thrumurthy S, Low DE (2012). Volume-outcome relationship in surgery for esophageal malignancy: systematic review and meta-analysis 2000–2011. J Gastrointest Surg.

[19] Bump RC, Mattiasson A, Bø K, Brubaker LP, DeLancey JO, Klarskov P, Shull BL, Smith AR (1996). The standardization of terminology of female pelvic organ prolapse and pelvic floor dysfunction. Am J Obstet Gynecol.

[20] Barber MD, Walters MD, Bump RC (2005). Short forms of two condition-specific quality-of-life questionnaires for women with pelvic floor disorders (PFDI-20 and PFIQ-7). Am J Obstet Gynecol.

[21] Rogers RG, Kammerer-Doak D, Villarreal A, Coates K, Qualls C (2001). A new instrument to measure sexual function in women with urinary incontinence or pelvic organ prolapse. Am J Obstet Gynecol.

[22] Wagemakers SH, van der Velden JM, Gerlich AS, Hindriks-Keegstra AW, van Dijk JFM, Verhoeff JJC (2019). A systematic review of devices and techniques that objectively measure patients' pain. Pain Physician.

[23] de Tayrac R, Faillie JL, Gaillet S, Boileau L, Triopon G, Letouzey V (2012). Analysis of the learning curve of bilateral anterior sacrospinous ligament suspension associated with anterior mesh repair. Eur J Obstet Gynecol Reprod Bio.

[24] Jeffery ST, Kortz BS, Muavha D, Stolwijk NN, Ras L, Roovers JWR (2018). Morbidity of a single incision transvaginal mesh to correct apical prolapse. J Minim Invasive Gynecol.

[25] Withagen MI, Vierhout ME, Hendriks JC, Kluivers KB, Milani AL (2011). Risk factors for exposure, pain, and dyspareunia after tension-free vaginal mesh procedure. Obstet Gynecol.

[26] Elmér C, Falconer C, Hallin A, Larsson G, Ek M, Altman D, Nordic Transvaginal Mesh Group (2012). Risk factors for mesh complications after trocar guided transvaginal mesh kit repair of anterior vaginal wall prolapse. Neurourol Urodyn.

[27] Altman D, Falconer C (2007). Perioperative morbidity using transvaginal mesh in pelvic organ prolapse repair. Obstet Gynecol.

[28] Parreira P, Maher CG, Steffens D, Hancock MJ, Ferreira ML (2018). Risk factors for low back pain and sciatica: an umbrella review. Spine J.

[29] Karppinen A, Ritvonen E, Roine R, Sintonen H, Vehkavaara S, Kivipelto L, Grossman AB, Niemelä M, Schalin-Jäntti C (2016). Health-related quality of life in patients treated for nonfunctioning pituitary adenomas during the years 2000–2010. Clin Endocrinol (Oxf).

